# Temporal evolution in caveolin 1 methylation levels during human esophageal carcinogenesis

**DOI:** 10.1186/1471-2407-14-345

**Published:** 2014-05-20

**Authors:** Zhe Jin, Liang Wang, Ziyi Cao, Yulan Cheng, Yan Gao, Xianling Feng, Si Chen, Huimin Yu, Wenjing Wu, Zhenfu Zhao, Ming Dong, Xiaojing Zhang, Jie Liu, Xinmin Fan, Yuriko Mori, Stephen J Meltzer

**Affiliations:** 1 Department of Pathology, The Shenzhen University School of Medicine, 3688 Nanhai Ave, Rm 703, Nanshan, Shenzhen 518060, Guangdong, People’s Republic of China; 2 Division of Gastroenterology, Department of Medicine, The Johns Hopkins University School of Medicine and Sidney Kimmel Comprehensive Cancer Center, Baltimore, MD, USA; 3 Shenzhen Key Laboratory of Micromolecule Innovatal Drugs, The Shenzhen University School of Medicine, Shenzhen, Guangdong, People’s Republic of China; 4 Shenzhen Key Laboratory of translational Medicine of Tumor, the Shenzhen University School of Medicine, Shenzhen, Guangdong, People’s Republic of China; 5 Laboratory of Chemical Genomics, School of Chemical Biology and Biotechnology, Peking University Shenzhen Graduate School, Shenzhen, Guangdong, People’s Republic of China; 6 Nanshan Hospital, Guangdong Medical College, Shenzhen, People’s Republic of China

**Keywords:** CAV1, Hypermethylation, EAC, ESCC

## Abstract

**Background:**

Esophageal cancer ranks eighth among frequent cancers worldwide. Our aim was to investigate whether and at which neoplastic stage promoter hypermethylation of *CAV1* is involved in human esophageal carcinogenesis.

**Methods:**

Using real-time quantitative methylation-specific PCR (qMSP), we examined *CAV1* promoter hypermethylation in 260 human esophageal tissue specimens. Real-time RT-PCR and qMSP were also performed on OE33 esophageal cancer cells before and after treatment with the demethylating agent, 5-aza-2’-deoxycytidine (5-Aza-dC).

**Results:**

*CAV1* hypermethylation showed highly discriminative ROC curve profiles, clearly distinguishing esophageal adenocarcinomas (EAC) and esophageal squamous cell carcinomas (ESCC) from normal esophagus (NE) (EAC *vs.* NE, AUROC = 0.839 and p < 0.0001; ESCC *vs.* NE, AUROC = 0.920 and p < 0.0001). Both *CAV1* methylation frequency and normalized methylation value (NMV) were significantly higher in Barrett’s metaplasia (BE), low-grade and high-grade dysplasia occurring in BE (D), EAC, and ESCC than in NE (all p < 0.01, respectively). Meanwhile, among 41 cases with matched NE and EAC or ESCC, *CAV1* NMVs in EAC and ESCC (mean = 0.273) were significantly higher than in corresponding NE (mean = 0.146; p < 0.01, Student’s paired t-test). Treatment of OE33 EAC cells with 5-Aza-dC reduced *CAV1* methylation and increased *CAV1* mRNA expression.

**Conclusions:**

*CAV1* promoter hypermethylation is a frequent event in human esophageal carcinomas and is associated with early neoplastic progression in Barrett’s esophagus.

## Background

Esophageal cancer ranks eighth among frequent cancers worldwide, with estimated over 480,000 new cases diagnosed and 400,000 deaths globally in 2008 [1]. There are two major histologic subtypes in esophageal cancer: esophageal adenocarcinoma (EAC), which is more prevalent in Western countries, with a rapid recent rate of increase in incidence; and esophageal squamous cell carcinoma (ESCC), which occurs at high frequencies in many developing countries, especially in Asia, and including China [2]. Since esophageal cancer exhibits highly aggressive behavior, with rapid progression to death [3,4], it is essential to gain a better understanding of the molecular events underlying these tumors In order to make further inroads into survival, it is important to discover novel early detection biomarkers and targets for chemoprevention or therapy.

Caveolae, which are small (50–100 nanometer) invaginations of the plasma membrane in many vertebrate cell types, are vital subcellular structures that regulate endocytosis, vesicular traffic, and signal transduction [5]. Caveolin-1 (CAV1), a cytoplasmic 22-kDa scaffold protein, is an essential component of caveolae [6]. In recent years, several studies have reported downregulation of CAV1 protein levels in several malignancies, including ovarian, breast [7-10], prostate [11], oral squamous cell [12] and lung cancer [13]. Prade *et al.* also showed that the majority of EACs exhibit loss of CAV1 expression in tumor *vs.* matched normal esophageal epithelia [14]. These results suggest that reduced CAV1 expression may represent a general characteristic of tumors, and that *CAV1* may inhibit tumor formation. Aberrant methylation of promoter CpG islands upstream of tumor suppressor genes is now well-established as a major mechanism of gene inactivation in tumorigenesis [15], including in ESCC and EAC [16-23]. Several of these aberrantly methylated genes appear to represent useful prognostic markers, as they precede and predict the progression of BE to EAC. Aberrant promoter methylation of *CAV1* is associated with inactivation of its expression in breast and colorectal cancers [24-27]. Therefore, we hypothesized that *CAV1* was inactivated via promoter hypermethylation in human esophageal cancers, and that hypermethylation of *CAV1* constituted an early event in the genesis of EAC.

## Methods

### Tissue samples

In the current study, 67 normal esophagi (NE), 60 Barrett’s metaplasias without dysplasia (BE), 19 low-grade (LGD) and 21 high-grade (HGD) dysplasias occurring in BE (D), 67 EACs, and 26 ESCCs were examined. Outcome data were derived from a comprehensive database maintained by the institution’s cancer registry and from patients’ medical records at the University of Maryland and Baltimore Veterans Affairs Medical Centers. All patients provided written informed consent under a protocol approved by the Institutional Review Boards at the University of Maryland and Baltimore Veterans Affairs Medical Centers, where all esophagogastroduodenoscopies were performed. Biopsies were taken using a standardized biopsy protocol, as previously described [17]. Research tissues were obtained from grossly apparent Barrett’s epithelium or from mass lesions in patients manifesting these changes at endoscopic examination, and histology was confirmed using parallel aliquots obtained at endoscopy. All biopsy specimens were stored in liquid nitrogen before DNA/RNA extraction. Clinicopathologic characteristics are summarized in Table 1.

**Table 1:** **Clinicopathologic characteristics and methylation status of** ***Caveolin 1*** **in human esophageal tissues**

	**Number of samples**	**Age (year) mean**	**NMV**		**Methylation status (cutoff 0.2)**			
**Clinical characteristics**			**Mean**	**p**	**Frequency**	**UM**	**M**	**p**
**Barrett’s segment**								
Short-segment ( <3 cm )	14	62.3	0.327	>0.05^§^	71.4%	4	10	> 0.05^‡^
Long-segment ( > = 3 cm )	16	62.8	0.439		81.3%	3	13	
**Histology**								
Normal esophagus	67	64.4	0.134		25.4%	50	17	
Barrett’s metaplasia	60	63.7	0.374	< 0.01*^/§^	81.7%	11	49	* < 0.01^†^
Dysplasia in Barrett’s esophagus	40	65.3	0.254	< 0.01*^/§^/ < 0.01^$/§^	60.0%	16	24	* < 0.01^†^/ ^$^ < 0.05^†^
Low-grade dysplasia	19	65.3	0.269	< 0.01*^/§^/ < 0.05^$/§^	57.9%	8	11	* < 0.01^†^/ ^$^ < 0.05^†^
High-grade dysplasia	21	65.2	0.240	< 0.01*^/§^/ < 0.01^$/§^	61.9%	8	13	* < 0.01^†^/ ^$^ > 0.05^†^
EAC	67	65.1	0.294	< 0.01*^/§^/ < 0.01^$/§^	65.7%	23	44	* < 0.01^†^/ ^$^ < 0.05^†^
Well differentiation	10	66.2	0.344	>0.05^§§^	80.0%	2	8	> 0.05^‡^
Moderate differentiation	24	66.1	0.29		58.3%	10	14	
Poor differentiation	22	65.5	0.297		63.6%	8	14	
Unknown	11	61	0.252		72.7%	3	8	
ESCC	26	62.5	0.326	< 0.01*^/§^	80.8%	5	21	* < 0.01^‡^
Well differentiation	3	61.7	0.307	>0.05^§§^	100.0%	0	3	> 0.05^‡^
Moderate differentiation	11	62.7	0.381		90.9%	1	10	
Poor differentiation	5	64.2	0.256		80.0%	1	4	
Unknown	7	61.1	0.299		57.1%	3	4	
**Stage of EAC patients**								
I	7	63	0.358	>0.05^§§^	71.4%	2	5	> 0.05^‡^
II	15	65.2	0.286		73.3%	4	11	
III	25	64.6	0.284		56.0%	11	14	
IV	7	66.3	0.242		28.6%	5	2	
**Lymph node metastasis in EAC patients**								
Negative	25	64.9	0.314	>0.05^§^	64.0%	9	16	> 0.05^†^
Positive	25	64.6	0.276		56.0%	11	14	
**Smoking status of EAC patients**								
Never	6	58.5	0.325	>0.05^§§^	83.3%	1	5	> 0.05^‡^
Former	24	68.5	0.276		62.5%	9	15	
Current	13	60.8	0.303		53.8%	6	7	
**Alcohol consumption of EAC patients**								
Never	16	65.3	0.285	>0.05^§§^	68.8%	5	11	> 0.05^‡^
Former	15	63	0.302		66.7%	5	10	
Current	10	65.7	0.315		60.0%	4	6	

EAC: esophageal adenocarcinoma; ESCC: esophageal squamous cell carcinoma; NMV: normalized methylation value; UM, unmethylated; M, methylated; ^§^Student's t test; *comparisons made to normal esophagus; *comparisons made to Barrett’s metaplasia; ^§§^Kruskal-Wallis test; ^†^Chi-square for independence test; ^‡^Fisher's exact test.

### Cell lines

The EAC (OE33) cell line was obtained from collaborators at the University of Michigan (Dr. David Beer). These cells were cultured in 47.5% RPMI 1640, 47.5% F-12 supplemented with 5% fetal bovine serum.

### DNA and RNA extraction

Genomic DNA was extracted from biopsies and cultured cells using a DNeasy Tissue Kit (Qiagen, Valencia, CA). Total RNA was isolated cultured cells using TRIzol reagent (Invitrogen, Carlsbad, CA). DNAs and RNAs were stored at -80°C before analysis.

### Bisulfite treatment and real-time quantitative methylation-specific PCR

DNA was treated with bisulfite to convert unmethylated cytosines to uracils prior to qMSP, as described previously [27]. Promoter methylation levels of *CAV1* were determined with the ABI 7900 Sequence Detection System (Applied Biosystems, Foster City, CA), using primers and probes as described previously [27]. A standard curve was generated using serial dilutions of CpGenome Universal Methylated DNA (CHEMICON, Temecula, CA). The normalized methylation value (NMV) was defined as follows: NMV = (*CAV1-S/CAV1-FM)/(ACTB-S/ACTB-FM*), where *CAV1-S* and *CAV1-FM* represent the methylation levels of *CAV1* in sample and universal methylated DNAs, respectively, while *ACTB-S* and *ACTB-FM* correspond to *ß*-*Actin* in sample and universal methylated DNAs, respectively [21].

### Real-time quantitative RT-PCR

To determine *CAV1* mRNA levels, one-step real-time quantitative reverse-transcriptase polymerase chain reaction (RT-PCR) was performed using a Qiagen QuantiTect Probe RT-PCR Kit (Qiagen, Hilden, Germany) and the ABI 7900 Sequence Detection System (Applied Biosystems, Foster City, CA). Primers and probes were the same as previously reported [27]. *ß*-*Actin* was used for normalization of data. A standard curve was generated using serial dilutions of qPCR Reference Total RNA (Clontech, Mountainview, CA). The normalized mRNA value (NRV) was calculated according to the following formula for relative expression of target mRNA: NRV *= (TarS/TarC)/(ACTB-S/ACTB-C),* where *TarS* and *TarC* represent levels of mRNA expression for the target gene in sample and control mRNAs, respectively, whereas *ACTB-S* and *ACTB-C* correspond to amplified *ß*-*Actin* levels in sample and control mRNAs, respectively [21].

### 5-Aza-dC treatment of esophageal cancer cell lines

To determine whether *CAV1* inactivation was due to promoter hypermethylation in esophageal cancer, OE33 EAC cells were subjected to 5-Aza-dC (Sigma, St. Louis, MO) treatment as previously described [21]. Briefly, 1 × 10^5^ cells/ml were seeded onto a 100 mm dish and grown for 24 h. Then, 1 ul of 5 mM 5-Aza-dC per ml of cells was added every 24 hours for 6 days. DNA and RNA were harvested on day 4.

### Data analysis and statistics

Receiver-operator characteristic (ROC) curve analysis [28] was performed using NMVs for the 67 EAC, 26 ESCC and 67 NE by Analyse-it software (Version 1.71, Analyse-it Software, Leeds, UK). Using this approach, the area under the ROC curve (AUROC) yielded optimal sensitivity and specificity to distinguish normal from malignant esophageal tissues, and corresponding NMV thresholds were calculated for *CAV1*. The cutoff value determined from this ROC curve was applied to determine the frequency of *CAV1* methylation in each tissue type included in the present study. For all other tests, Statistica (version 6.1; StatSoft, Inc., Tulsa, OK) was used. Differences with p < 0.05 were deemed significant.

## Results and discussion

### *CAV1* promoter hypermethylation in different esophageal tissues

**Figure 1:**
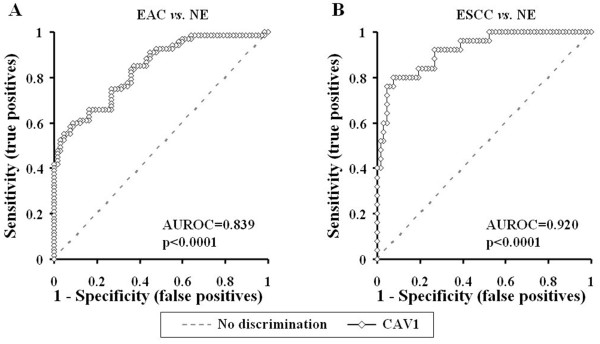
**Receiver-operator characteristic (ROC) curve analysis of normalized methylation value (NMV).** ROC curve analysis of *CAV1* NMVs of normal esophagus (NE) *vs*. esophageal adenocarcinoma (EAC) **(A)**, and NE *vs.* esophageal squamous cell carcinoma (ESCC) **(B)***.* The area under the ROC curve (AUROC) conveys this biomarker’s accuracy in distinguishing NE from EAC and from ESCC in terms of its sensitivity and specificity.

Promoter hypermethylation of the *CAV1* gene was analyzed in 67 NE, 60 BE, 40 D (19 LGD and 21 HGD), 67 EAC and 26 ESCC samples. All assays in this study were performed in duplicate format, and data showed reproducible and concordant results. *CAV1* promoter hypermethylation showed highly discriminative ROC curve profiles, which clearly distinguished both EAC (p < 0.0001) and ESCC (p <0.0001) from NE. AUROC of EAC *vs.* NE and ESCC *vs.* NE were 0.839 and 0.920, respectively. ROC curves with corresponding AUROCs for *CAV1* of EAC *vs.* NE and ESCC *vs.* NE are displayed in Figure 1.

**Figure 2:**
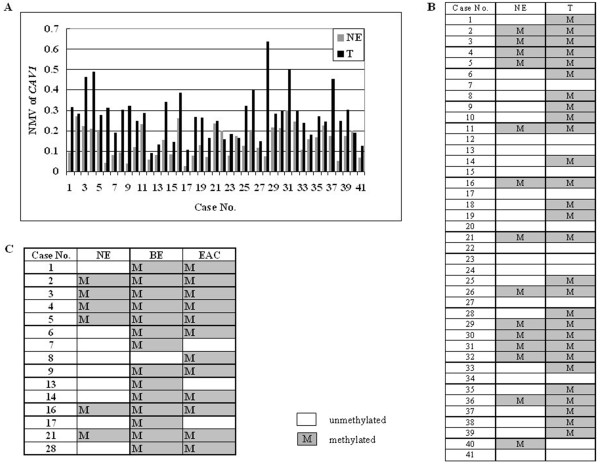
**Methylation status of *CAV1 *in matched esophageal tissue samples. A**, NMVs of *CAV1* in 41 patients with matched NE and esophageal cancer (T, EAC or ESCC). **B**, methylation status of *CAV1* in 41 cases with corresponding NE and T. **C**, methylation status of *CAV1* in 15 cases with corresponding NE, BE and EAC.

**Figure 3:**
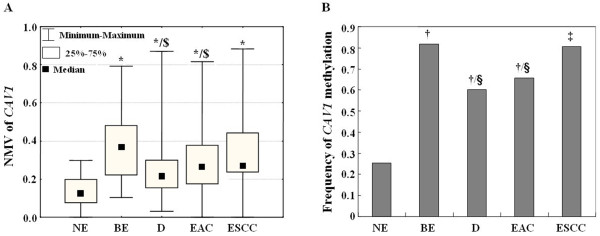
**Methylation profiles of *CAV1 *in different esophageal tissue samples. A**, The mean NMV of *CAV1* was significantly higher in ESCC, EAC, D, and BE than in NE, and in BE than in D and EAC. **B**. The frequency of *CAV1* hypermethylation was significantly higher in BE, D, EAC and ESCC than in NE, and in BE than in D and EAC. NE: normal esophagus; BE: Barrett’s metaplasia; D: Dysplasia in BE; EAC: esophageal adenocarcinoma; ESCC: esophageal squamous cell carcinoma; *: Student’s t test, p < 0.01 (comparisons made to NE); $: Student’s t test, p < 0.01 (comparisons made to BE); †: Chi-square for independence test, p < 0.01 (comparisons made to NE); ‡: Fisher’s exact test, p < 0.01 (comparisons made to NE); §: Chi-square for independence test, p < 0.05 (comparisons made to BE).

The cutoff NMV for *CAV1* (0.2) was chosen from ROC curves to maximize sensitivity and specificity. Mean NMV and frequency of *CAV1* hypermethylation for each tissue type are shown in Table 1. The mean NMV of *CAV1* was significantly higher in ESCC (0.326), EAC (0.294), D (0.254), HGD (0.240), LGD (0.269) and BE (0.374) than in NE (0.134; p < 0.01, Student’s t-test). The frequency of *CAV1* hypermethylation was increased in BE (81.7%), D (60%), and EAC (65.7%) *vs.* NE (25.4%; p < 0.01, p < 0.01 and p < 0.01, respectively; Chi-square for independence test). *CAV1* was hypermethylated in 21 (80.8%) of 26 ESCCs. There were no significant differences between EAC and ESCC in mean *CAV1* NMV (0.294 *vs.* 0.326; p > 0.05) or hypermethylation frequency (65.7% *vs.* 80.8%, p > 0.05). Among 41 cases with matched NE and T (EAC or ESCC), *CAV1* NMVs in T (mean = 0.273) were significantly higher than in corresponding NE (mean = 0.146; p < 0.01, Student’s paired t-test; Figure 2A and B). Among 15 cases with corresponding NE, BE and EAC, one (No.8) was methylated only in EAC, three (No.7, 13 and 17) were methylated only in BE, five (No.1, 6, 9, 14 and 28) were methylated in both BE and EAC, and the remaining six were methylated in NE, BE and EAC simultaneously (Figure 2C). In addition, both *CAV1* mean NMV and hypermethylation frequency were significantly higher in BE (0.374, 81.7%) than in D (0.254, 60%) and EAC (0.294, 65.7%; p < 0.01 and p < 0.05, respectively; Figure 3).

No significant associations were observed between *CAV1* promoter hypermethylation and patient age, survival (data not shown), smoking or alcohol consumption status, BE segment length, tumor stage or lymph node metastasis, histologic tumor differentiation, or histologic type of esophageal carcinoma (Table 1).

### *CAV1* methylation and mRNA levels in OE33 cells after 5-Aza-dC treatment

**Figure 4:**
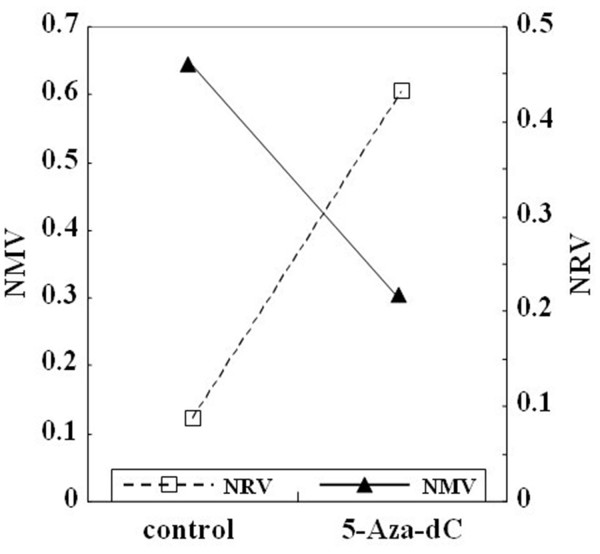
***CAV1 *methylation and mRNA expression in OE33 cells after treatment with 5-aza-2’-deoxycytidine (5-Aza-dC).** After 5-Aza-dC treatment, the NMV of *CAV1* was diminished, while the normalized mRNA value (NRV) of *CAV1* was increased.

OE33 cells were subjected to demethylation by 5-Aza-dC treatment. On day 4 after 5-Aza-dC treatment, the NMV of *CAV1* was diminished, while *CAV1* mRNA levels were increased (Figure 4).

*CAV1* has already been previously reported to have tumor suppressor activity *via.* inhibiting cell proliferation and/or metastesis in several human cancers [29-32]. CAV1 down-regulation has been reported in many types of cancer, including breast, lung, oral and esophagus [9,10,12-14]. These results suggest that low expression of CAV1 may represent a general characteristic or even a requirement of transformed cells in many kinds of carcinogenesis. Potential mechanisms underlying this suppression of expression include posttranscriptional and epigenetic changes, such as aberrant DNA methylation [26,27,33]. In the current study, we systematically investigated hypermethylation of the *CAV1* gene promoter in primary human esophageal lesions of differing histological types and grades. Our results demonstrate that *CAV1* promoter hypermethylation occurs frequently in both human EAC and ESCC (Table 1). *CAV1* NMVs in T were significantly higher than those in corresponding NE (p < 0.01, Student’s paired t-test) in 41 cases with corresponding NE and T (Figure 2). Moreover, hypermethylation of the *CAV1* promoter was significantly more frequent in premalignant lesions, such as BE and D, as well as in EAC, than in NE (Table 1). There was no significant association between *CAV1* promoter hypermethylation and histological subtype of esophageal carcinoma (EAC *vs.* ESCC). These results suggest that hypermethylation of CAV1 may represent an early epigenetic event in these subjects, that the frequency of this epigenetic event increases during esophageal carcinogenesis, and that this event is highly prevalent in human esophageal cancers.

Barrett’s carcinogenesis is a multistep process comprising genetic and epigenetic alterations in tumor suppressor genes, cell cycle-regulatory genes, and genes essential for cell–cell adhesion [34,35]. Progressive accumulation of gene alterations is postulated to underly the transition of normal squamous epithelium to BE [36]. Many previous studies, focused on promoter hypermethylation of candidate genes for esophageal carcinomas, have shown staged growth in methylation frequency from nondysplastic esophageal squamous cell mucosa to BE and finally to EAC [15,37,38]. Interestingly, it has been suggested that *CAV1* acts as a tumor modulator in a tissue type- and stage-dependent manner by binding several different proteins involved in different signal transduction [6,39-42]. Recently, we reported a preponderance of hypomethylation over hypermethylation events during the epigenomic program of BE pre-progression by comparing global DNA methylation profiles of two groups of BE patients, termed ‘progressors’ and ‘non-progressors’ [16]. Although the frequency of *CAV1* hypermethylation in this study increased in parallel with esophageal carcinoma progression, the mean NMV and frequency of *CAV1* hypermethylation were higher in BE than in D and EAC, and these differences were significant by Student’s t test (*i.e.,* there was an inverse correlation between *CAV1* hypermethylation and Barrett’s-associated esophageal neoplastic progression) (Figure 3). Taken together, these data suggested that the *CAV1* promoter is relatively hypomethylated in EAC and D *vs.* BE, implying that, at least in part, this event represents an early part of the temporal program of Barrett’s-associated esophageal neoplastic progression. Two previous studies demonstrated that expression of CAV1 was elevated in ESCC compared to corresponding normal tissues, and its elevation was associated with malignant progression and poor survival [43,44]. These inconsistent results may have been due to different analytic methods used, ethnic groups studied, and smaller sample sizes in the previous studies.

In accordance with previous findings [11,45,46], we observed that methylation of *CAV1* in EAC cell lines was associated with silenced or reduced expression of *CAV1* mRNA. In this study, reversal of methylation and restoration of *CAV1* expression were induced in OE33 cells by 5-Aza-dC treatment (Figure 4). Restoration of *CAV1* mRNA expression due to 5-Aza-dC treatment is consistent with the interpretation that DNA hypermethylation silences the *CAV1* gene. Although 5-Aza-dC or its derivatives have shown potential as therapeutic anticancer drugs [47-49], relatively hypomethylation of *CAV1* in EAC and D *vs.* BE in the current study, and together with previous data on the re-expression of CAV1 in advance cancerf, would make CAV1 not an ideal molecular target for anti-cancer therapy involving demethylation in EAC patients.

## Conclusions

The current study indicates that hypermethylation of the *CAV1* promoter, leading to gene silencing, is a common event in human esophageal cancer and occurs early during Barrett’s-associated EAC. These results provide a basis for further research on *CAV1* as a potential biomarker for the early diagnosis, classification, stratification and prognostication of esophageal cancers.

## Abbreviations

5-Aza-dC: 5-aza-2’-deoxycytidine; BE: Barrett’s esophagus; CAV1: Caveolin-1; EAC: Esophageal adenocarcinomas; ESCC: Esophageal squamous cell carcinomas; NE: Normal esophagus; NMV: Normalized methylation value; qMSP: Real-time quantitative methylation-specific PCR; ROC: Receiver-operator characteristic.

## Competing interests

The authors declare that they have no competing interests.

## Authors’ contributions

ZJ and SJM designed the study. ZJ, LW and ZC wrote the main manuscript text. YC, YG, XF, SC, HY, WW, ZZ, MD, XZ, JL, XF and YM analyzed and interpreted the data. All authors reviewed the manuscript. All authors read and approved the final manuscript.

## Authors’ information

Corresponding author: Zhe Jin, Department of Pathology, The Shenzhen University School of Medicine, 3688 Nanhai Ave, Rm 703, Nanshan, Shenzhen, Guangdong, People’s Republic of China 518060; Phone: 086-0755-86671904; Fax: 086-0755-86671906; email address: zhejin1995@yahoo.com Co-Correspondence: Stephen J. Meltzer, Division of Gastroenterology, Department of Medicine, Johns Hopkins University School of Medicine. 1503 E. Jefferson Street, Rm. 112, Baltimore, MD, USA 21231; Phone: 01-410-502-6071; Fax: 01-410-502-1329; email address: smeltzer@jhmi.edu

## Pre-publication history

The pre-publication history for this paper can be accessed here:

http://www.biomedcentral.com/1471-2407/14/345/prepub
